# Cytological Assessments and Transcriptome Profiling Demonstrate that Evodiamine Inhibits Growth and Induces Apoptosis in a Renal Carcinoma Cell Line

**DOI:** 10.1038/s41598-017-12918-y

**Published:** 2017-10-03

**Authors:** Xiao-Long Yuan, Peng Zhang, Xin-Min Liu, Yong-Mei Du, Xiao-Dong Hou, Sen Cheng, Zhong-Feng Zhang

**Affiliations:** 1 Tobacco Research Institute of Chinese Academy of Agricultural Sciences, Qingdao, China; 2 Shanghai Tobacco Group Company Limited, Shanghai, China

## Abstract

Chinese medicines are an important source of secondary metabolites with excellent antitumour activity. *Evodia rutaecarpa*, from the family Rutaceae, exhibits antitumour activity. Evodiamine (EVO), which was isolated from the fruit of *E*. *rutaecarpa*, exhibits robust antitumour activity. However, the antitumour mechanism of EVO remains unclear. In this study, we assessed the growth-inhibiting effect of EVO on two renal carcinoma cell lines. We found that EVO could change the morphology and decrease the viability and proliferation of cells in a time- and concentration-dependent manner *in vitro*. In addition, transcriptome analysis indicated that EVO can modulate the transcriptome of Caki-1 cells. In total, 7,243 differentially expressed genes were found, among which 3,347 downregulated genes and 3,896 upregulated genes were mainly involved in cell migration, apoptosis, cell cycle, and DNA replication. Furthermore, we demonstrated that EVO can cause apoptosis, arrest cells in the G2/M phase, and regulate the expression of apoptosis- and cell cycle-related genes in Caki-1 cells. Our study reveals the anticancer effects of EVO using cellular and molecular data, and indicates the potential uses of this compound as a resource to characterize the antitumour mechanisms of *E*. *rutaecarpa*.

## Introduction

Renal cell carcinoma (RCC) is one of the most serious cancers in adults, accounting for ~90% of all renal malignancies^1^. The annual incidence of RCC is continually increasing^2^. Because of its highly invasive and metastatic characteristics, the initial diagnosis is often delayed. For example, nearly 30% of patients have distant metastases and 25% have locally advanced disease when they are initially diagnosed^3–5^. The survival rate of patients with metastatic RCC can be as low as 9.5%^6^. Because RCC is insensitive to radiotherapy and chemotherapy^7^, therapy with targeted agents (e.g., interleukin-2, temsirolimus, or pazopanib) has proven to be superior compared to other treatments^8,9^. However, not all of treatments can produce better therapeutic effects or achieve complete responses^10–13^. Therefore, an urgent need exists to develop novel therapeutics that overcome resistance to the currently administered anticancer drugs for RCC.

Evodiamine (EVO) is an indoloquinazoline alkaloid isolated from fruit of *Evodia rutaecarpa* ^14^. For millennia, it has been considered an effective Chinese medicine that can be used for the treatment of gastropathy, hypertension, and eczema. In the previous decade, increasing evidence has revealed that EVO exhibits various biological effects, including antibacterial, antiobesity, and antinociceptive activities^15–17^. Recently, it has been established that EVO exhibits antitumour activity against gastric adenocarcinoma, colorectal carcinoma, and breast cancer cells^18–21^. Recently, Wu and colleagues investigated the anticancer mechanism of EVO against renal carcinoma cells at the cellular and protein levels. Their results showed that EVO induces renal carcinoma cell apoptosis via disruption of the mitochondrial membrane potential, resulting from JNK- and PERK-mediated phosphorylation of the Bcl-2 protein^22^. However, the mechanism by which EVO exerts its anticancer effects on renal carcinoma cells requires further research.

In this study, cytological experiments and a transcriptome profiling study were performed to reveal the anticancer mechanism of EVO. First, a series of experiments investigating the viability and proliferation of human renal carcinoma 786-O cells and Caki-1 cells and the human renal epithelial cell line HK-2 was performed under EVO treatment. Then, a transcriptome profiling study was used to analyse EVO-regulated genes and signalling pathways responsible for growth and apoptosis using RNA sequencing. In addition, we verified the molecular mechanisms through which EVO inhibits growth and induces apoptosis using ultrastructural observations, flow cytometry analysis, and qPCR.

## Materials and Methods

### Cell culture and EVO treatments

Three cell lines (human renal carcinoma cell lines (786-O and Caki-1 cells) and the human renal epithelial cell line HK-2) were purchased from the Chinese Academy of Sciences Committee on Type Culture Collection Cell Bank (Shanghai, China). The 786-O and Caki-1 cells were cultured in RPMI 1640 medium (Invitrogen, Carlsbad, CA, USA) supplemented with 10% foetal bovine serum (FBS; Gibco, Carlsbad, CA, USA) and the HK-2 cells were maintained in keratinocyte serum-free medium (Gibco BRL, Grand Island, NY, USA) supplemented with 50 μg/ml bovine pituitary extract (BPE) and 5 ng/ml recombinant epithelial growth factor. All cells were maintained in a humidified atmosphere with 95% air and 5% CO_2_ at 37 °C.

EVO with a purity of more than 99% was obtained from J&K Scientific (Beijing, China). Before use, 20 mg of EVO powder was dissolved in dimethyl sulphoxide (DMSO) to prepare a 30.36 mg/ml stock solution. For EVO treatment and antitumour analysis, cells were treated with serially diluted doses of EVO for 24, 48, or 72 h. All control cells were cultured in medium containing 0.2% DMSO. A separate evaluation of the effects of 0.2% DMSO in this study did not reveal any significant differences between DMSO-treated and untreated cells.

### Cell viability analysis

The Cell Counting Kit-8 (CCK-8) colorimetric method was used to determine the sensitivity of cells to EVO. Briefly, the three cell lines were inoculated into 96-well plates at 3.0 × 10^4^ cells per well. All treatments were performed in triplicate and in parallel. After 24 h, supernatants were replaced, and cells were exposed to different concentrations of EVO (0, 5, 10, 20, 40, 60, 80, and 100 μg/ml). After 24, 48, and 72 h, 10 μL of 5 g/L CCK-8 solution was added to each well and then the cells were incubated at 37 °C for 1.5 h in the dark. A microplate spectrophotometer reader (Multickan GO, Thermo Scientific, Waltham, MA, USA) was used to measure the absorbance of cells per well at 490 nm, which was used to calculate the percent inhibition of cell growth for each group.

### Colony formation assay

Cell proliferation in response to EVO was estimated using the colony formation assay. The concentration of human renal carcinoma cells (786-O and Caki-1 cells) was adjusted to 1 × 10^5^ cells/ml per well. Cells were cultured in six-well plates and exposed to various concentrations of EVO for 48 h. The medium was replaced regularly and all cultures were incubated for 2 weeks. Colonies were fixed using 70% ethanol solution and then stained with Giemsa. The surviving fraction was calculated and compared to that of the control to estimate the clonogenic efficiency.

### Flow cytometry analysis

Caki-1 cells exposed to 40 μg/ml EVO for 24, 48, and 72 h were collected at a concentration of 1 × 10^6^ cells/ml. Cells were washed three times using phosphate-buffered saline (PBS). For cell cycle analysis, fixed cells were stained with propidium iodide (PI) solution (20 μg/mL) containing 0.1% Triton X-100 (Sigma–Aldrich, St. Louis, MO, USA) and 100 μg/mL RNase A (Fermentas International Inc., Burlington, ON, Canada). For Annexin V-FITC/PI analysis, cells were stained with FITC-Annexin V Apoptosis Detection Kit I (BD Pharmingen) following the manufacturer’s instructions. A FACScan flow cytometer was used to detect the stained cells (at least 10,000 cells), and CXP analysis software (BD Biosciences, San Jose, CA, USA) was used to analyse the flow cytometry data.

### Fluorescence microscopy

The morphology of Caki-1 cells was observed using Hoechst 33258, acridine orange/ethidium bromide (AO/EB), and transferase-mediated deoxyuridine triphosphate-biotin nick end labelling (TUNEL) staining, as described previously^23–26^. Briefly, Caki-1 cells were first incubated for 24 h and then exposed to 40 μg/ml EVO for 24, 48, and 72 h for assessments via fluorescence microscopy. For AO/EB staining, cells were trypsinized and resuspended in 0.1 ml of serum-free RIPA 1640 medium. Then, the cells were stained with 4 μL of AO/EB solution (100 μg/ml; Sigma–Aldrich) at 37 °C for 15 min. The remaining Caki-1 cells were fixed with 70% ethanol for Hoechst 33258 and TUNEL staining. The magnitude of DNA damage was examined via TUNEL assay, using a commercial kit (Roche, Basel, Switzerland) following the manufacturer’s instructions. Subsequently, the cells were stained with 4 ng/ml 4′,6-diamidino-2-phenylindole (DAPI) or 1 μg/ml Hoechst 33258 solution (Sigma–Aldrich) at 4 °C for 5–10 min. Stained cells in each group were observed using a confocal laser scanning microscope (Leica Microsystems, Hessen Wetzlar, Germany).

### Cellular ultrastructure examination

Cellular ultrastructure was investigated using transmission electron microscopy (TEM), as described previously^21^. Briefly, Caki-1 cells exposed to 40 μg/ml EVO for 24, 48, and 72 h were harvested as mentioned above. The cells were fixed with 2% glutaraldehyde (containing 0.1 M sucrose and 0.2 M sodium cacodylate) overnight at 4 °C, followed by 10 g/l osmium tetroxide. The samples were dehydrated and embedded in epoxy resin. Ultra-thin sections were observed using an H700 TEM (Hitachi, Tokyo, Japan).

### RNA extraction

Total RNA of cells was extracted using a Cell RNA Kit (Omega Bio-Tek, Inc., Norcross, GA, USA) according to the manufacturer’s instructions. The integrity of the RNA was determined on 1% agarose gels. RNA quality and concentration were determined using a NanoPhotometer spectrophotometer (IMPLEN, Westlake Village, CA, USA).

### Library construction for DGE sequencing

To explore the mechanism underlying the effects of EVO on human renal carcinoma cells, Caki-1 cells exposed to 40 μg/ml EVO for 48 h or without EVO treatment were collected for RNA extraction. For library construction, 5 μg RNA of each sample was used. Libraries were constructed using a NEBNext Ultra RNA Library Prep Kit for Illumina (NEB, Ipswich, MA, USA) according to the manufacturer’s instructions. When constructing the libraries, a unique barcode was added to each sample. First, mRNA was isolated from total RNA with poly(T)-oligo-conjugated magnetic beads. After purification, the mRNA was fragmented and used to synthesize the first strand using random hexamer primers and M-MuLV reverse transcriptase (RNase H). Subsequently, the second strand of cDNA was generated using DNA polymerase I and RNase H. Before adenylation of the 3ʹ ends of cDNA, exonuclease/polymerase was used to repair cohesive ends. Then, the specific adaptor for the Illumina library was added to each sample. cDNA fragments with a length of 150–200 bp were selected and purified using an AMPure XP system (Beckman Coulter, Beverly, MA, USA). Finally, these libraries were amplified and then subjected to purification. The quality and quantity of the final libraries were assessed on an Agilent Bioanalyzer 2100.

### Analysis of differentially expressed genes

Differential expression analysis between the experimental and control groups was performed using the DESeq R package (1.18.0). Fragments per kilobase of transcript (FPKM) were used to measure gene expression levels. P-values were generated for each gene and the adjusted P-value was set at 0.05 for the subsequent analysis.

### Functional annotation and GO/KEGG enrichment analysis

For functional annotation of differentially expressed genes, gene ontology (GO) enrichment and Kyoto Encyclopedia of Genes and Genomes analyses were implemented using the GOseq R package and KOBAS software, respectively^27–30^.

### Real-time quantitative PCR validation

Total RNA extraction was performed as described above. Briefly, 1 μg of total RNA was reverse-transcribed for validation of differentially expressed genes. In total, 16 genes were amplified using custom-designed primers. Two other pairs of primers for glyceraldehyde-3-phosphate dehydrogenase (GAPDH) and β-actin were used to amplify the internal reference genes, as shown in Supplementary File S5. The qPCR was performed using LightCycle 480. The reaction volume was 20 μl and included the following: 10 μl of SYBR mix, 0.6 μl of each primer, 2 μl of cDNA template, and 6.8 μl of RNA-free water. The thermocycling program was as follows: 95 °C for 5 min, followed by 45 cycles of 95 °C for 10 s, 57 °C for 10 s, and 72 °C for 20 s. Relative gene expression levels were calculated using the 2^−ΔΔCt^ method^31^.

### Statistical analysis

In our study, triplicate experiments were performed in parallel. For data analysis, the SPSS 21.0 software package (Chicago, IL, USA) was used to detect statistically significant differences between means. Compared with negative controls, differences were considered statistically significant if P < 0.05.

## Results

### EVO reduced cell viability and impaired renal carcinoma cell proliferation

**Figure 1:**
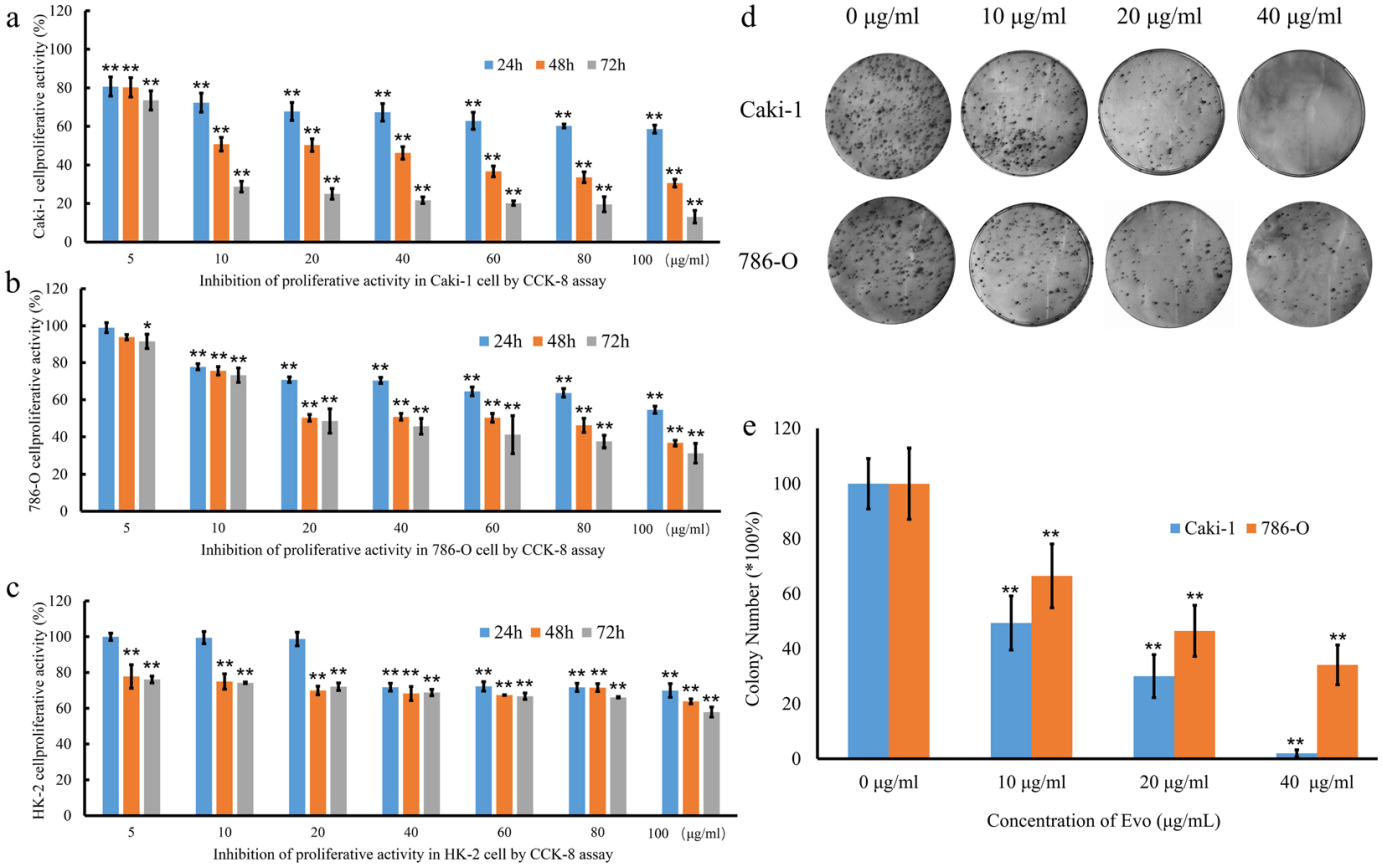
Effect of EVO on indicated renal carcinoma cell lines. (**a**–**c**) Inhibitory effect of EVO on three cell lines (renal carcinoma cell lines Caki-1 and 786-O and the human renal epithelial cell HK-2). The survival rate of Caki-1 and 786-O cells after treatment with EVO (**d**,**e**) based on a colony formation assay (n = 5; ^*^P < 0.05, ^**^P < 0.01).

First, we investigated the inhibitory activity of EVO against the proliferation of three cell lines (human renal carcinoma cell lines (786-O and Caki-1 cells), and the human renal epithelial cell line HK-2) using the CCK-8 assay. The three cell lines were exposed to increasing concentrations (5–100 μg/mL) of EVO for different incubation times (24, 48, and 72 h). We found that EVO decreased the viability of these three cell lines in a time- and concentration-dependent manner (Fig. 1a,b). However, the sensitivity to EVO was different among the three cells. The most remarkable effect was detected for Caki-1 cells, whereas a minimal growth-inhibiting effect was observed for HK-2 cells (Fig. 1c). The IC_50_ was 23.707 μg/mL at 48 h in Caki-1 cells. In addition, the colony-formation assay demonstrated that EVO significantly inhibited the proliferation of Caki-1 and 786-O cells compared with the control group (Fig. 1d,e), which was consistent with the results of the CCK-8 assay. Based on this finding, Caki-1 cells were used in subsequent experiments.

### EVO-induced modulation of Caki-1 cells at the transcriptional level

**Figure 2:**
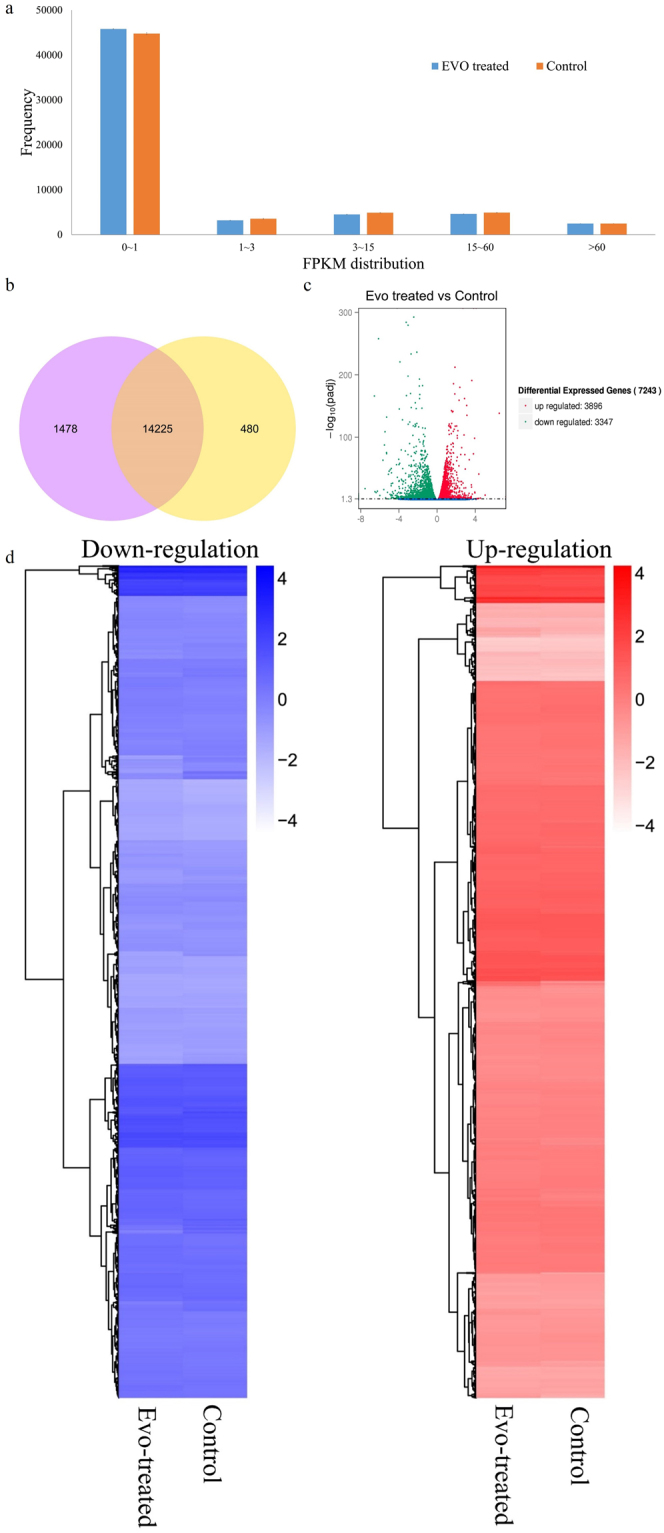
Overview of genes differentially expressed between EVO-treated Caki-1 cells and untreated cells. (**a**) Column diagram of gene expression values. (**b**) Venn diagram of the genes differentially expressed between EVO-treated Caki-1 cells and untreated cells. (Cc Volcano diagram of genes differentially expressed between EVO-treated Caki-1 cells and untreated cells. (**d**) Heatmaps of upregulated and downregulated genes in EVO-treated Caki-1 cells and untreated cells.

To explore the mechanism underlying the antitumour effects of EVO, a transcriptional profiling analysis between EVO-treated Caki-1 cells and untreated cells was performed. In total, 27,366,108 reads in the EVO-treated group and 30,288,463 reads in the control group were generated via RNA-seq. These reads were mapped to the human genome, and the unique mapping rates reached 80%, which represented 14,705 and 15,703 expressed genes, respectively. Then, FPKM was used to quantify gene expression levels. The differences in gene expression levels between the treated and control groups are shown in Fig. 2a and Table S1. These results indicated that 480 and 1478 genes were uniquely expressed in the EVO-treated and control groups, respectively (Fig. 2b). According to the standard of P < 0.05 and fold change (FC) > 2, 7243 differentially expressed genes were found, among which 46.21% (3347) were downregulated and 53.79% (3896) were upregulated (Fig. 2c). Comparisons between the treated and control groups were performed using heatmaps. The standard normal distribution of expression signals for every gene was used for normalization (Fig. 2d).

### Functional analysis of differentially expressed genes

**Figure 3:**
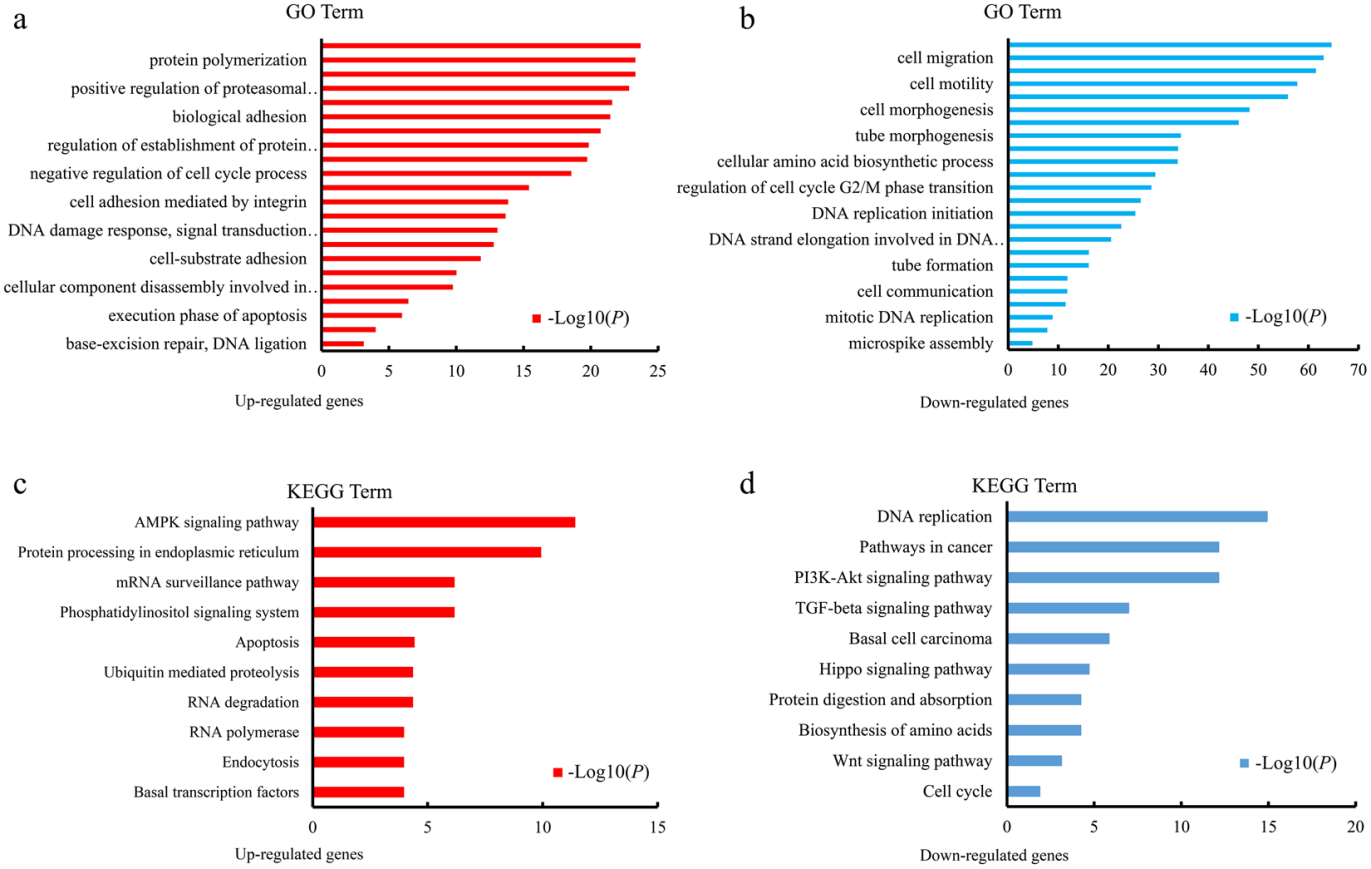
GO and KEGG analysis of differentially expressed genes. (**a**,**b**) Upregulated and downregulated genes linked with GO enrichment terms. (**c**,**d**) Upregulated and downregulated genes in KEGG pathway analysis.

To understand the function of genes differentially expressed between the treated and control groups, GO category and KEGG pathway analyses for the differentially expressed genes were conducted. The enrichment of GO categories for these genes showed that the upregulated genes were mainly involved in protein polymerization, cell adhesion, negative regulation of the cell cycle, apoptosis, and the DNA damage response (Fig. 3a), and the downregulated genes were mainly related to cell migration, cell motility, cell and tube morphogenesis, cell cycle, and DNA replication (Fig. 3b). Analysis of the metabolic pathways that these differentially expressed genes participated in showed that the most enriched upregulated pathways were involved in mitogen-activated protein kinase (MAPK) signalling, endoplasmic reticulum, phosphatidylinositol signalling, apoptosis, and endocytosis (Fig. 3c), whereas the most enriched downregulated gene pathways included DNA replication, phosphatidylinositol-3 kinases/protein kinase B (PI3K/Akt) signalling, transforming growth factor-β (TGF-β) signalling protein digestion and absorption, and the cell cycle (Fig. 3d). The effects of EVO were most closely associated with the pathways of apoptosis (Figure S1), cell cycle (Figure S2), and DNA replication (Figure S3), all of which can decrease cell proliferation capacity and viability. These findings indicate that EVO might modulate Caki-1 cell biological processes by inhibiting the cell cycle and inducing apoptosis.

### EVO induced G2/M-phase arrest in Caki-1 cells

**Figure 4:**
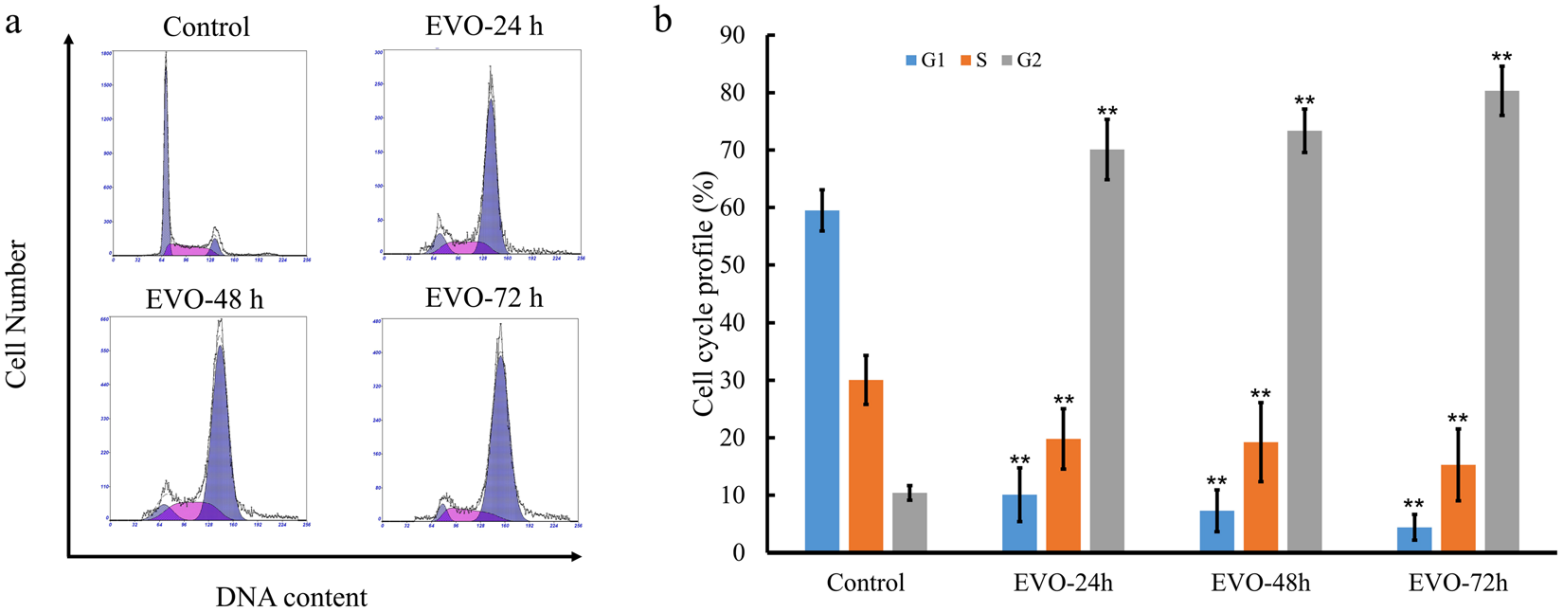
Cell cycle profiling of Caki-1 cells exposed to EVO. (**a**) Cell cycle distribution of EVO-treated Caki-1 cells. (**b**) Cell cycle profile of EVO-treated Caki-1 cells.

To assess the effects of EVO on the cell cycle, Caki-1 cells were treated with 40 μg/ml EVO for 24, 48, and 72 h, respectively, and evaluated via flow cytometry. As shown in Fig. 4a,b, cell population in the G2 phase significantly increased upon treatment with EVO by 10.41%, 70.1%, 73.36%, and 80.31% compared with that in the control group. Additionally, EVO-treated cells exhibited a reduction in the population in S phase (30.08%, 19.8%, 19.25%, and 15.28%) and G1 phase (59.51%, 10.1%, 7.29%, and 4.41%). These findings indicate that EVO inhibits Caki-1 cell proliferation by inducing cell cycle arrest at the G2/M phase.

### EVO induces apoptosis

**Figure 5:**
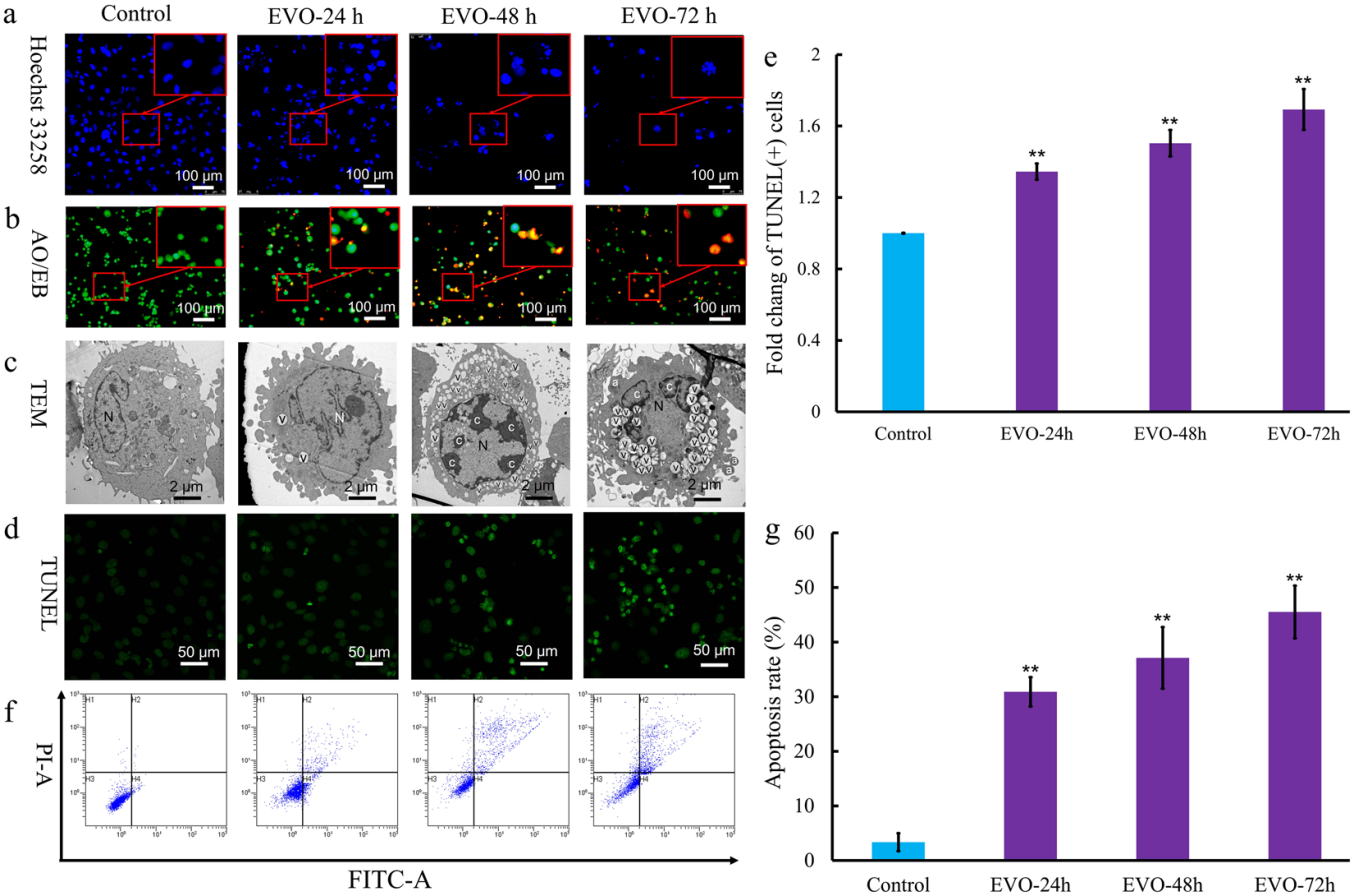
EVO induced apoptosis in Caki-1 cells. (**a**) Apoptosis-related morphological changes were detected by staining cells with Hoechst 33258. Apoptotic cells were defined as those with blue-stained nuclei that exhibited a fragmented/condensed appearance (red arrow). Original magnification, × 200. (**b**) Elevated plasma membrane permeability was detected by staining cells in the AO/EB double-staining assay (white arrow). Original magnification, × 200. (**c**) Ultrastructural changes in Caki-1 cells were detected via TEM. a, apoptotic body; c, condensed chromatin; mv, microvillus; N, nucleus; v, vacuole. (**d**) The 3ʹ OH ends of fragmented DNA of apoptotic cells were labelled in the TUNEL assay and viewed under a fluorescence microscope. Original magnification, × 400. (**e**) Bar graph showing the quantification of TUNEL-positive Caki-1 cells. (**f**) Annexin V-FITC and PI reactivity was used to detect and quantify apoptosis induced by EVO. The percentage of cells undergoing apoptosis was determined via flow cytometry. Mean values are shown with the SD and significant differences between the EVO-treated and untreated groups are indicated as ^*^(P < 0.05) or ^**^(P* < *0.01).

To further explore the role of apoptosis in EVO treatment of Caki-1 cells, morphological changes were observed via fluorescence microscopy and TEM. We found that Caki-1 cells exhibited time-dependent apoptosis-like morphological changes (Fig. 5) after exposure to 40 μg/ml EVO at times ranging from 24 to 72 h. The observed morphological features included bright staining (Fig. 5a), condensed chromatin (Fig. 5a), elevated permeability (Fig. 5b), microvillus disappearance (Fig. 5c), ultrastructural disorganization (Fig. 5c), vacuolation (Fig. 5c), and apoptotic body formation (Fig. 5c). Moreover, apoptosis was investigated via TUNEL assay and flow cytometry to quantitatively analyse apoptotic cells. TUNEL was used to evaluate fragmented DNA in apoptotic cells. After EVO treatment for 24, 48, and 72 h, cells were stained with TUNEL and analysed via fluorescence microscopy (Fig. 5d,f). The proportions of apoptotic cells were 30.9%, 37.12%, and 45.51% after treatment with EVO ranging from 24 to 72 h, respectively, compared with 3.36% for the control group (Fig. 5e,g).

### Validation of the expression of apoptosis- and cell cycle-associated genes

To validate the differentially expressed genes identified by RNA-Seq analysis, qRT-PCR was performed to determine the expression levels of 16 genes associated with apoptosis and the cell cycle. We measured the mRNA transcript levels of genes involved in apoptosis, including interleukin-1 (*IL1*), tumour necrosis factor receptor type 1-associated death domain protein (*TRADD*), interleukin-1 receptor-associated kinase (*IRAK*), cytochrome C (*cyto-c*), apoptosis-inducing factor (*AIF*), *PI3K*, endonuclease G (*ENDOG*), and protein kinase A (*PKA*), and the cell cycle, including tumour protein (*p53*), cell-division cycle protein (*Cdc14*), *TGF-β*, mothers against decapentaplegic homolog 2 (*Smad2*), growth arrest and DNA damage protein 45 (*GADD45*), checkpoint kinase 1 (*Chk1*), mini-chromosome maintenance 5 (*Mcm5*), and mini-chromosome maintenance 6 (*Mcm6*).

**Figure 6:**
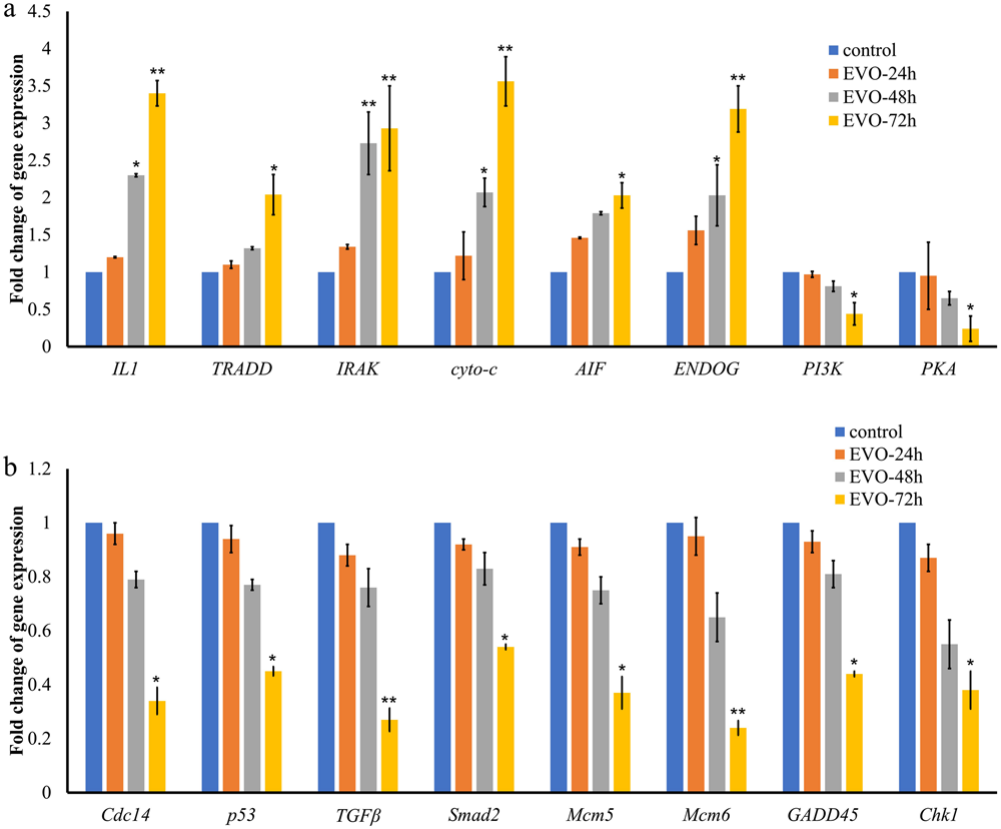
Validation of expression levels of genes related to apoptosis and the cell cycle. (**a**) Increased gene expression of apoptosis-related genes induced by EVO. (**b**) Decreased gene expression of cell cycle-related genes induced by EVO.

We observed a significant increase in the expression of genes involved in the induction of apoptosis (*IL1*, *TRADD*, *IRAK*, *cyto-c*, *AIF*, and *ENDOG*), whereas there was a reduction in the expression of genes involved in the inhibition of apoptosis (*PI3K* and *PKA*). Additionally, we detected a significant reduction of *p53*, *Cdc14*, *TGF-β*, *Smad2*, *GADD45*, *Chk1*, *Mcm5*, and *Mcm6* transcript levels; these genes play important roles in cell cycle arrest (Fig. 6b). The relative expression levels of these genes were consistent with the RNA-Seq findings (Table S2).

## Discussion

Currently, the incidence and mortality of renal carcinoma are increasing globally, and existing treatments have not yet substantially prolonged survival^32,33^. The development of novel therapeutic agents for renal carcinoma is urgently needed^32,34^. The antibacterial, antiobesity, and antinociceptive effects of EVO have been investigated in previous studies^14–21^. However, the mechanism by which EVO exerts anticancer effects on renal carcinoma cells requires further research^22^. Herein, we provide evidence that EVO suppresses proliferation and induces apoptosis in renal carcinoma cells by affecting multiple cell signalling molecules based on cytology experiments and a transcriptome profiling study.

To characterize the antitumour mechanism of EVO, we first investigated cell viability by adding EVO to cultures of human renal carcinoma cell lines (Caki-1 and 786-O) and the human renal epithelial cell line HK-2. EVO decreased the viability of 786-O and Caki-1 cells, which is in line with the previous finding that EVO decreased the viability of various renal carcinoma cells^22^. The most obvious antitumour effects of EVO on Caki-1 cells were observed in the CCK-8 assay after comparison of cell viability. Additionally, the cell proliferation findings, based on a colony formation assay, were consistent with our cell viability findings. Preliminary tests showed that EVO could decrease the cell viability. To identify the mechanism that accounts for the EVO-induced antitumour effect, genes differentially expressed between EVO-treated and untreated groups were identified based on transcriptome analysis. In total, 7,243 differentially expressed genes were observed, and their functions were further analysed by GO and KEGG analysis. We found that EVO could affect Caki-1 cells by affecting the following biological processes: apoptosis, the cell cycle, translation, nuclear division, and cell division. Thus, EVO influenced the expression of genes related to apoptosis and cell cycle. The effects of EVO on Caki-1 cells were similar to those observed for polysaccharides on a non-small cell lung cancer cell line^35^.

Changes in the expression levels of cycle-related genes have often been reported to be related to DNA damage^36^. Our TUNEL assay results indicated that EVO could induce DNA damage over time; moreover, DNA damage has been found to be induced by EVO treatment of other renal carcinoma cells (i.e., 786-O cells and ACHN cells)^22^. The fidelity of replication is affected by DNA damage, and severe DNA damage could cause cells to undergo cell cycle arrest^37,38^. Additionally, our findings indicated that EVO could arrest the cell cycle of Caki-1 cells at the G2/M phase, which is consistent with previous research showing that EVO could induce G2/M arrest in human A498 RCC cells^22^. Moreover, our RNA-seq and qRT-PCR analysis showed that *p53*, *Cdc14*, *TGF-β*, *Smad2*, *GADD45*, *Chk1*, *Mcm5*, and *Mcm6* were all downregulated in EVO-treated Caki-1 cells. Among the identified genes, *p53* plays a vital role in regulation of the cell cycle by controlling the expression of *GADD45* ^39^_,_ one of the activators in the MAPK signalling pathway. *TGF-β* is a key regulator of cell fate and transmits its signals via *Smad2* ^40^. Minichromosome maintenance protein complex (MCM) is a eukaryotic DNA helicase complex that is essential for DNA replication and partially contributes to the chromatin association and phosphorylation of Chk1^41^. *p53* and *CDC14* can arrest cells at the G2/M phase through *PI3K* and *Chk1* ^42^. The downregulation of these genes was closely related to the cell cycle, leading to decreased proliferation and viability of Caki-1 cells, which is in accordance with the results of the TUNEL and cell cycle assays. These cellular and transcriptional level data all support the hypothesis that EVO can arrest the cell cycle of renal carcinoma cells. It is well established that a cell may undergo apoptosis if it cannot progress through the G2/M checkpoint^43^. Therefore, the cell cycle arrest, combined with reduced viability and proliferative capacity, imply that EVO may have an apoptosis-inducing effect on Caki-1 cells *in vitro*.

Induction of cancer cell apoptosis is one of the main modes of cancer treatment^44,45^. Apoptotic cells present characteristics such as elevated plasma membrane permeability, phosphatidylserine (PS) externalization, and apoptotic body formation^46–48^. Therefore, plasma membrane permeability, PS orientation, DNA integrity, and the ultrastructure of EVO-treated Caki-1 cells were determined via AO/EB staining, flow cytometry analysis of Annexin-V/PI staining, PI staining, and TEM, respectively, to detect the effects of EVO on apoptosis. Our findings indicate that EVO could induce elevation of plasma membrane permeability in Caki-1 cells, which is similar to the effects previously observed for antitumour agents such as *Schisandra chinensis* polysaccharide and quercetin^49,50^. EVO could also induce PS externalization along with typical apoptotic-like ultrastructural changes, such as structural disorganization, vacuolation, and apoptotic body formation in Caki-1 cells. Moreover, we observed that the transcriptional levels of *IL-1* mRNA transcripts increased, and the production of IL-1 can induce growth reduction and apoptosis by regulation of the downstream substrate *IRAK* ^51^. In addition, we observed that EVO could induce apoptosis by increasing the expression of genes involved in NF-κB-mediated apoptosis (i.e., *TRADD*) and mitochondrial apoptosis (i.e., *AIF*, *ENDO-G*, and *cyto-c*) pathways^52^^,^^53^. In contrast, genes that block the apoptotic death of cancer cells were all shown to be downregulated^54^. Overall, our data support the conclusion that EVO prominently regulated the expression of these genes and induced apoptosis, which underlies its antitumour effects.

The study findings reveal how EVO exerts its effects against renal carcinoma cells through cell viability and genome-wide differential gene expression analyses. As EVO is a new antitumour modality, the correlation between these findings *in vitro* and *in vivo* toxicology assessments should be further studied.

## Electronic supplementary material

Supplementary Information

Dataset 1

Dataset 2

Dataset 3

Dataset 4

Dataset 5
