# Supplementary material for: Cytological Assessments and Transcriptome Profiling Demonstrate that Evodiamine Inhibits Growth and Induces Apoptosis in a Renal Carcinoma Cell Line
Source: Sci Rep. 2017 Oct 3;7:12572. doi: 10.1038/s41598-017-12918-y (PMC5626725; doi:10.1038/s41598-017-12918-y)
Supplement: Supplementary file 1 — Supplementary Information [file 41598_2017_12918_MOESM1_ESM.pdf]

Cytological Assessments and Transcriptome Profiling Demonstrate that Evodiamine Inhibits Growth and Induces Apoptosis in a Renal Carcinoma Cell Line

Xiao-Long Yuan[1], Peng Zhang[1], Xin-Min Liu[1], Yong-Mei Du[1], Xiao-Dong Hou[1], Sen Cheng[2] & Zhong-Feng Zhang[1,*]

[1] Tobacco Research Institute of Chinese Academy of Agricultural Sciences, Qingdao, China. [2] Shanghai Tobacco Group Company Limited, Shanghai, China. [*]Correspondence and requests for materials should be addressed to Z.-F. Z. (email: zhangzhongfeng@caas.cn)

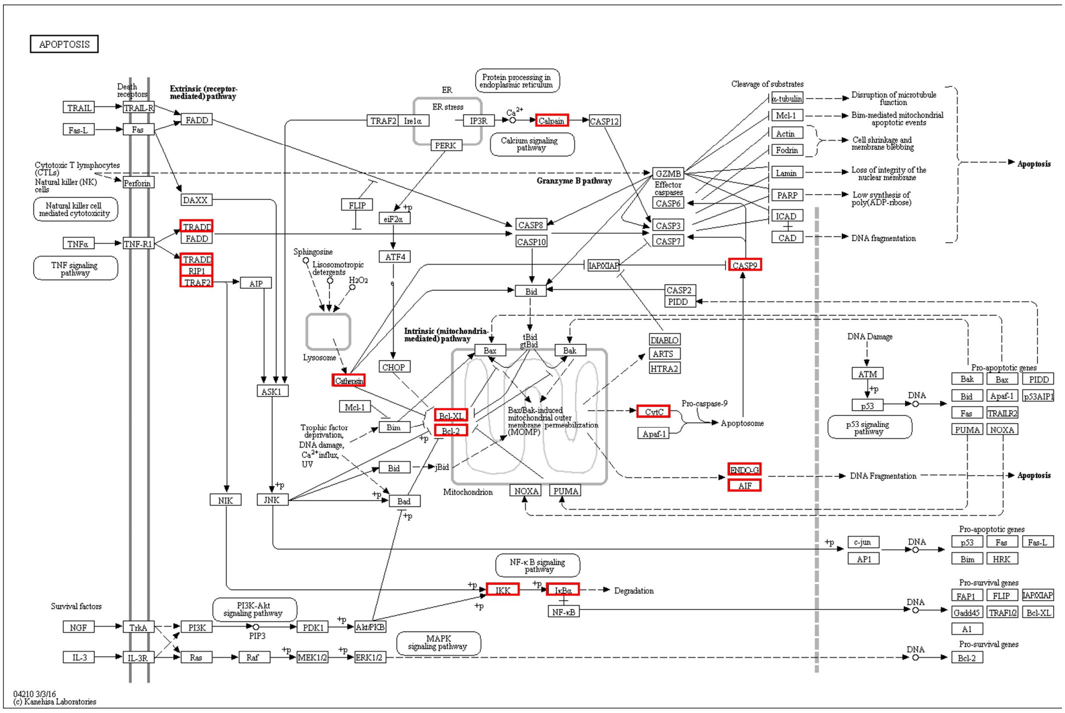

Figure S1 Schematic diagram of differentially expressed genes involved in apoptosis by KEGG[1-3]. (http://www.kegg.jp/kegg-bin/show_pathway?map04210)

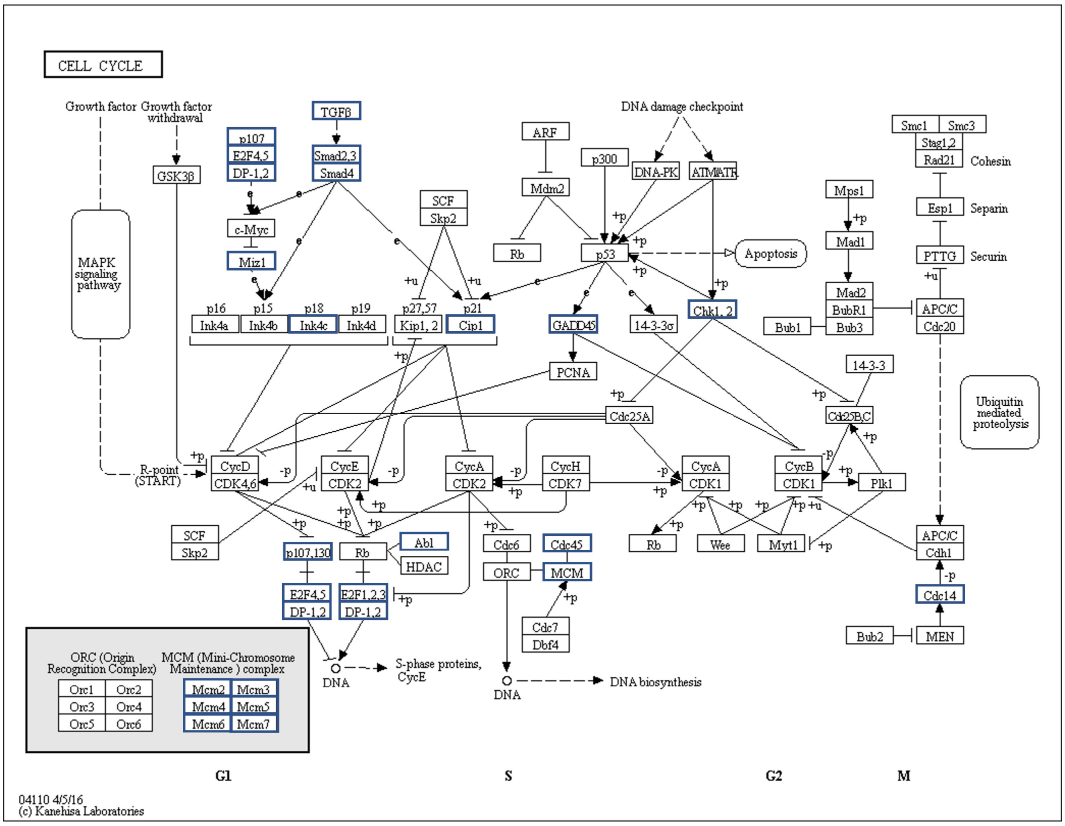

Figure S2 Schematic diagram of differentially expressed genes involved in the cell cycle by KEGG[1-3]. (http://www.kegg.jp./kegg-bin/show_pathway?map04110)

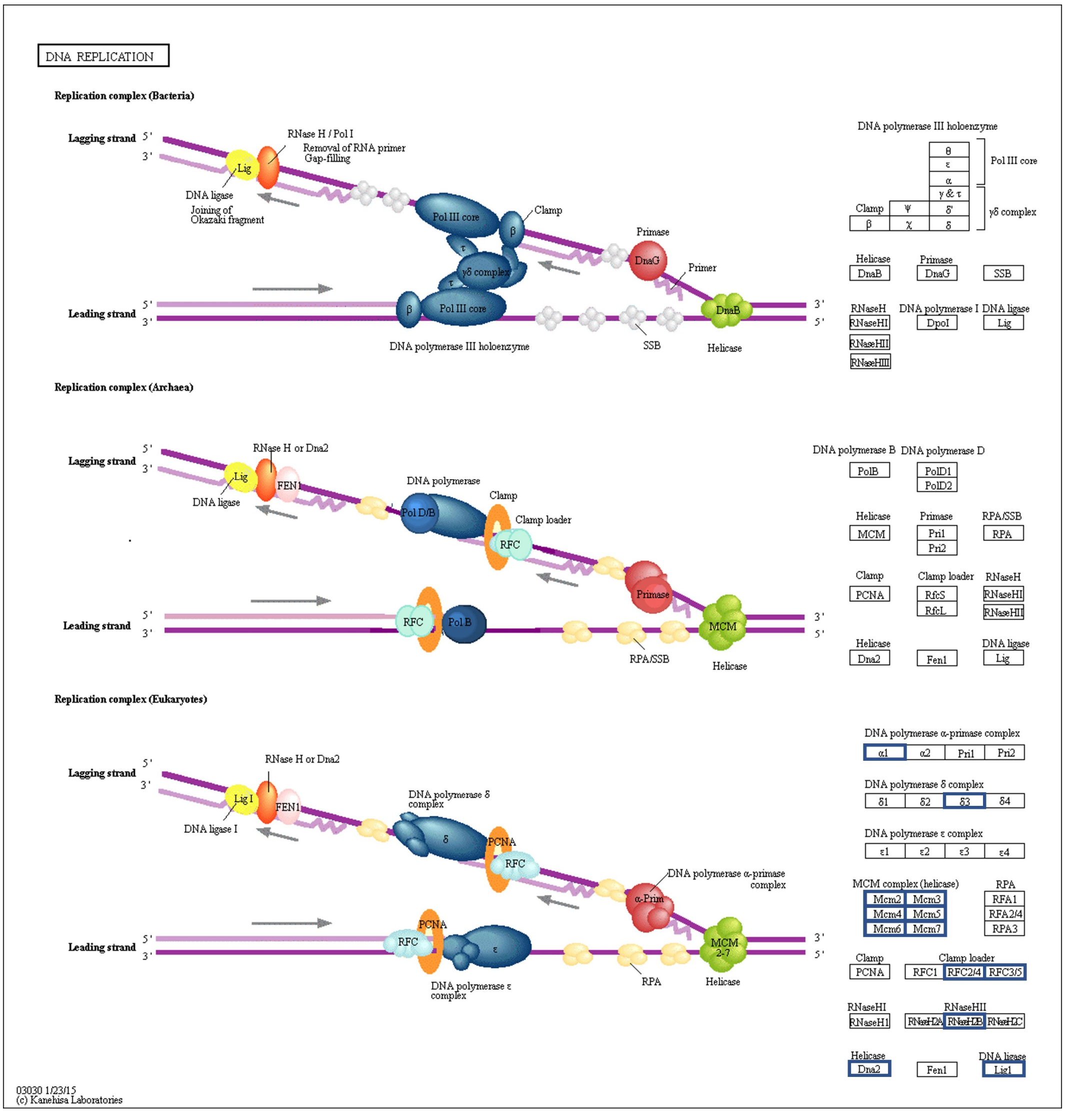

Figure S3 Schematic diagram of differentially expressed genes involved in the DNA replication by KEGG[1-3]. (http://www.kegg.jp./kegg-bin/show_pathway?map03030)

References

1 Kanehisa, M. *et al*. KEGG: new perspectives on genomes, pathways, diseases and drugs. *Nucleic Acids Research* **45**, D353-D361 (2017)

2 Kanehisa, M. *et al*. KEGG as a reference resource for gene and protein annotation. *Nucleic Acids Research* **44**, D457-D462 (2016).

3 Kanehisa, M. & Goto, S. KEGG: Kyoto Encyclopedia of Genes and Genomes. *Nucleic Acids Research* **28**, 27-30 (2000).
